# A case report of isolated lymphadenopathy revealing localized leishmanial lymphadenopathy in an asthenic 25-year-old man

**DOI:** 10.1097/MD.0000000000003932

**Published:** 2016-07-22

**Authors:** Quentin Hurlot, Judith Fillaux, Camille Laurent, Antoine Berry, Paul Hofman, Bruno Marchou, Pierre Delobel, Pierre Brousset, Guillaume Martin-Blondel

**Affiliations:** a Department of Pathology, Institut Universitaire du Cancer; b Department of Parasitology, Toulouse University Hospital; c INSERM, Centre de Physiopathologie Toulouse-Purpan, Toulouse; d Department of Pathology, Nice University Hospital, Nice; e Department of Infectious and Tropical Diseases, Toulouse University Hospital; f CRCT INSERM U1037, Toulouse; g Laboratoire d’Excellence Toulouse Cancer LABEX-TOUCAN, France.

**Keywords:** amphotericin B, diagnosis, leishmaniasis, lymphadenopathy

## Abstract

**Background:**

Visceral leishmaniasis (VL) is endemic in large areas of the tropics, the subtropics, and the Mediterranean basin. Besides classical VL presentation, exceptional cases of a limited form of VL have been reported. Here we describe the challenges of diagnosis and management of this intriguing entity.

**Case summary:**

A 25-year-old French Caucasian man presented with marked asthenia that had lasted 6 months and was strictly isolated except for a 2-cm left cervical lymphadenopathy. The rest of the clinical examination and extensive biological exploration were unremarkable.

Histological examination of the cervical lymphadenopathy showed a reactive lymphoid hyperplasia with granulomatous organization associated with small particles in the cytoplasm of epithelioid histiocytes and giant cells evocative of Leishman–Donovan bodies. Polymerase chain reaction (PCR) performed on the tissue confirmed the presence of *Leishmania donovani/infantum* DNA. Direct examination of a bone marrow aspiration, together with blood and bone marrow PCR, did not find other evidence for VL. Serology for leishmaniasis was unreactive. Extensive work-up for other causes of granulomatous lymphadenitis was negative. A diagnosis of localized leishmanial lymphadenopathy was made. Intravenous liposomal amphotericin B (20 mg/kg in five infusions) was initiated and well tolerated. Asthenia disappeared promptly and the patient fully recovered.

**Conclusion:**

Localized lymph node enlargement because of leishmanial infection should be included in the differential diagnosis of lymphadenopathy of unknown origin in patients who stayed or visited, even a long time ago and for a short period, endemic areas for leishmaniasis such as the Mediterranean basin. Fine-needle aspiration cytology and/or PCR for *Leishmania sp* of the lymphadenopathy might contribute to the diagnosis. A low-dose liposomal amphotericin B treatment might be effective, and deserves further study.

## 1 Introduction

Visceral leishmaniasis (VL) is a zoonotic vector-borne disease caused by the *Leishmania donovani* protozoa complex *(L donovani and Leishmania infantum*).^[1]^ VL is endemic in large areas of the tropics, subtropics, and the Mediterranean basin and can occur in travelers coming back from affected countries. The complex parasite–host interactions that predispose some individuals to developing the disease or to controlling the infection influence a spectrum of clinical manifestations ranging from asymptomatic to fatal visceral infections.^[2]^ Here we report an unusual case of VL presenting as an isolated cervical lymphadenopathy in an immunocompetent host.

## 2 Case presentation

**Figure 1:**
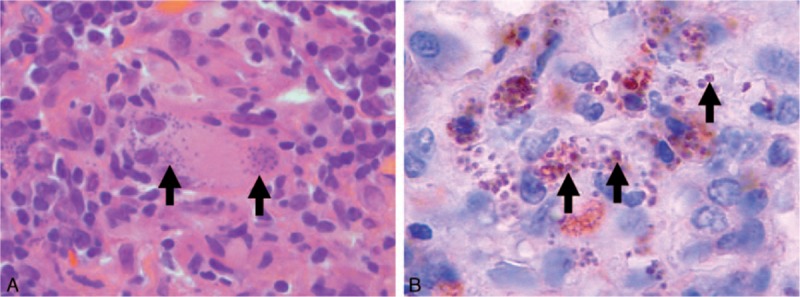
Histopathology of the cervical lymphadenopathy. (A) Hematoxylin and eosin coloration (magnification ×1000) of the lymphadenopathy showing granulomatous organization with giant cells and epithelioid histiocytes. Arrows show Leishman–Donovan bodies. (B) Immunohistochemistry of the lymphadenopathy using a monoclonal anti-Leishman antibody (magnification ×1200), demonstrating leishmanial parasites with their nuclei (arrows).

A 25-year-old French Caucasian man presented in December 2014 with marked asthenia that had lasted 6 months. He had no fever, weight loss, or night sweats and was asymptomatic except for this profound asthenia. He had no past medical history. This patient was born and lived in Paris, France, and had moved 2 years before Toulouse, in the southwestern part of France. He traveled to Egypt for 1 week in 2009, spent 1 month in China and Vietnam in July 2013, and 2 weeks in Corsica in September 2013. The clinical examination was unremarkable except for a firm and mobile 2 cm left cervical lymphadenopathy without inflammatory signs. In particular, he had no other lymphadenopathy, nor any hepatosplenomegaly, and the otorhinolaryngology examination was normal. Laboratory blood tests showed normal cell count, creatinine, C-reactive protein, hepatic enzyme, and lactate dehydrogenase levels. Erythrocyte sedimentation rate, as well as serum protein electrophoresis, was normal. Serologies for human immunodeficiency virus (HIV), syphilis, and *Bartonella henselae* were unreactive. Serology for toxoplasmosis showed IgG without IgM. A computed tomography scan of the neck, chest, abdomen, and pelvis showed no other lymphadenopathy, and no evidence for sarcoidosis, tuberculosis, or cancer. The cervical lymphadenopathy was biopsied. The histologic examination of the lymphadenopathy revealed a reactive lymphoid hyperplasia with granulomatous organization. Hematoxylin and eosin (Fig. 1A), *Periodic Acid–Schiff* and Grocott (not shown) colorations showed small particles in the cytoplasm of epithelioid histiocytes and giant cells evocative of Leishman–Donovan bodies. Polymerase chain reaction (PCR) performed on the biopsy confirmed the presence of *L donovani/infantum* DNA^[3]^ and was negative for *Toxoplasma gondii*. Immunohistochemistry using a monoclonal antibody p19–11 raised against the *Leishmania* homologue of receptors for activated C-kinase was also positive (diluted 1:80,^[4]^Fig. 1B). Immunohistochemistry for *T gondii* (*T gondii* Ab-1 Rabbit polyclonal antibody) and HIV (anti-HIV-1 p24 antibody), and in situ hybridization for Epstein–Barr virus-encoded RNA, were negative. Direct examination of a bone marrow aspiration, together with blood and bone marrow PCR, did not find other evidence for VL. Serology for leishmaniasis was unreactive by *indirect fluorescent assay* (*vircell leishmania indirect fluorescent antibody IgG L infantum*) and immunochromatography (BioRad-IT Leish “*L infantum*”), but homemade *L infantum*-specific Western blot showed 14- and 18-kDa bands.^[5]^ Extensive work-up for other causes of granulomatous lymphadenitis, including PCR in blood, stool, and saliva for *Tropheryma whipplei*, was negative. A diagnosis of localized leishmanial lymphadenopathy was made. Intravenous liposomal amphotericin B (20 mg/kg in 5 infusions) was initiated and well tolerated. Asthenia disappeared promptly and the patient fully recovered.

## 3 Discussion

After being transmitted by the bite of phlebotomine sand flies, leishmanial amastigotes disseminate through the lymphatic and vascular systems and infect the reticulo-endothelial system, resulting in infiltration of the bone marrow, liver, spleen, and lymph nodes.^[1]^ Approximately 0.2 to 0.4 million new VL cases occur each year, with an overall case fatality rate of 10%. The most common presentation of VL includes signs of persistent systemic infection (high-grade fever, asthenia, anorexia, and weight loss) and of parasitic invasion of the blood and reticulo-endothelial system (lymphadenopathy, hepatomegaly, splenomegaly, pancytopenia, and hypergammaglobulinemia). VL symptoms often persist for several weeks to months before patients either seek medical care or die from bacterial co-infections, massive bleeding, or severe anemia. Liposomal amphoterin B is used as first-line treatment in Europe and the United States.^[6]^ However, asymptomatic infections are far more frequent than symptomatic VL cases in immunocompetent hosts, demonstrating that many people infected with visceral leishmanial species develop an effective immune response and do not manifest the clinical disease.^[7]^ In between, rare cases of a limited form of VL presenting as isolated lymphadenopathy without other evidence of VL have been described as localized leishmanial lymphadenopathy.^[8]^ The clinical presentation of this emerging entity has been recently defined in 17 consecutive patients presenting with localized leishmanial lymphadenopathy during a large outbreak of *L infantum* VL that occurred in Spain.^[9]^ Localized leishmanial lymphadenopathy exclusively affects immunocompetent hosts. As in the case described here, patients presented with indolent lymphadenopathy affecting mainly the cervical area (65%) without fever or systemic symptoms. Diagnosis can be made by fine-needle aspiration cytology of the lymphadenopathy, showing granulomatous lymphadenitis with leishmanial parasites. PCR for *L donovani/infantum* DNA on aspiration might also be a useful tool. Serological testing was not reliable, as half of the patients in this study had negative results. Although no consensus on therapeutic management of localized leishmanial lymphadenopathy has been formulated,^[6]^ patients were usually treated for VL. Regarding that form of the disease, in the Spanish study, 2 patients displayed lymphadenopathies affecting different areas, and 1 had clinical and biological signs evocative of early stages of typical VL, suggesting that localized leishmanial lymphadenopathy might evolve toward a systemic disease.^[9]^ However, treatments received were heterogeneous, and liposomal amphoterin B was used, with total dose varying from 10 to 30 mg/kg. Overall, the outcome was favorable, regardless of the dose used. Because patients suffering from localized leishmanial lymphadenopathy are not immunocompromised and the disease is still limited to the lymph node, the use of lower total doses of liposomal amphoterin B (10 mg/kg) has been advocated.

## 4 Conclusion

Localized lymph node enlargement because of leishmanial infection should be included in the differential diagnosis of lymphadenopathy of unknown origin in patients who stayed or visited, even a long time ago, endemic areas for leishmaniasis such as the Mediterranean basin (Table 1). Fine-needle aspiration cytology and/or PCR for *Leishmania sp* of the lymphadenopathy might contribute to the diagnosis. A low-dose liposomal amphotericin B treatment might be effective and deserves further study.

**Table 1:**
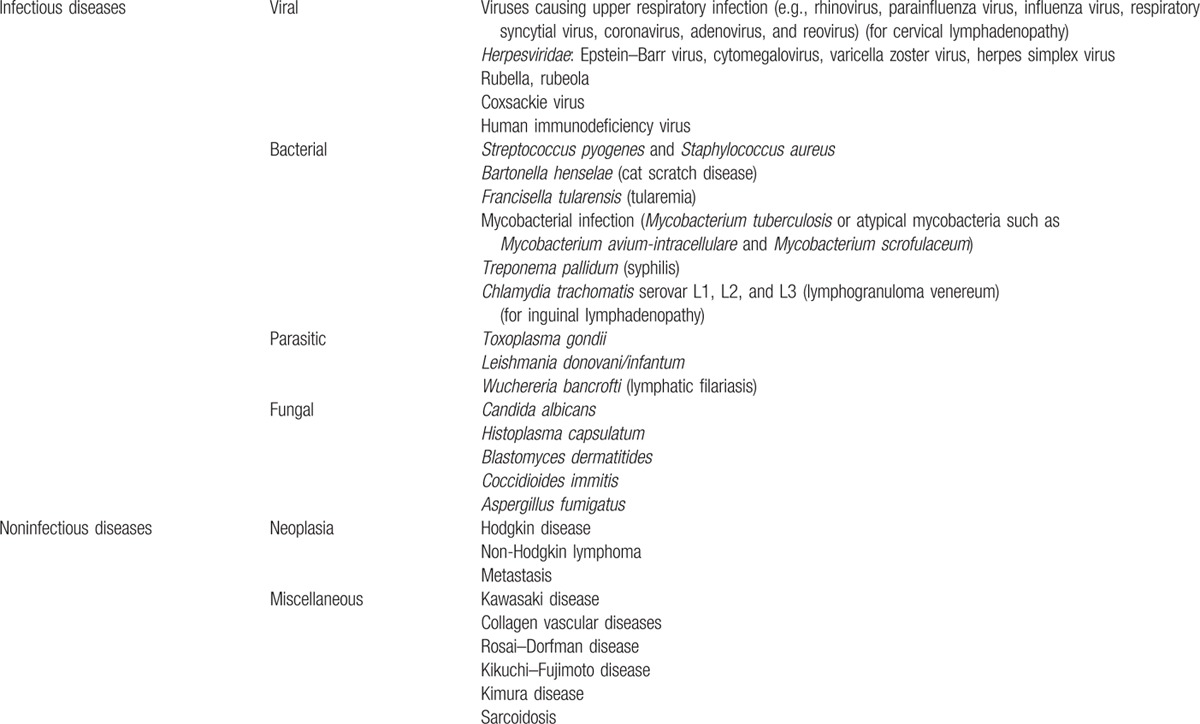
Main causes of localized lymphadenopathy (enlargement of a single node or multiple contiguous nodal regions, adapted from^[10]^).

## Acknowledgments

The authors would like to thank David and Virginia Clark for their kind help in English editing.
